# Targeting *Bifidobacterium animalis* alleviates high-fluoride exposure-induced kidney injury in mice

**DOI:** 10.1186/s13568-026-02031-7

**Published:** 2026-04-08

**Authors:** Yu Chen, Ning Sun, Baoxing Gan, Yuhao He, Jiuyang Luo, Kangcheng Pan, Yan Zeng, Bo Jing, Dong Zeng, Xueqin Ni

**Affiliations:** 1 Animal Microecology Institute, College of Veterinary Medicine, Sichuan Agricultural University, No. 211 Huimin Road, Wenjiang District, Chengdu, Sichuan China; 2 School of Public Health, Southern Medical University, Guangzhou, Guangdong China

**Keywords:** High-fluoride exposure, Probiotics, Kidney injury, Metabolomics, Metagenomic

## Abstract

In areas with high fluoride concentrations in drinking water, residents may consume excessive fluoride, which may increase the risk of renal impairment. Although accumulating evidence suggests that probiotics may exert renoprotective effects, support for probiotic interventions against fluoride-associated renal injury remains limited, and the effects appear to be strain dependent. In a prolonged exposure model, mice received sodium fluoride in drinking water (25 or 50 ppm) for 56 weeks, after which renal function was assessed and metagenomic profiling was performed. Mice exposed to varying fluoride concentrations developed renal injury, and the relative abundance of *Bifidobacterium animalis* was significantly correlated with markers of renal function. A short-term fluoride-exposure model (sodium fluoride, 24 mg/kg/day for 8 weeks, by gavage) was used to evaluate the renal protective effect of *Bifidobacterium animalis* GY007. Supplementation with GY007 significantly reduced renal injury markers, including β2-microglobulin (β2-MG) and lipocalin 2 (LCN2). GY007 reduced pro-inflammatory cytokines interleukin-6 (IL-6) and tumor necrosis factor-α (TNF-α), increased anti-inflammatory interleukin-10 (IL-10), and alleviated oxidative stress. Transmission electron microscopy (TEM) analysis indicated that GY007 improved mitochondrial morphology in damaged renal tissue. Further analyses showed that GY007 improved mitochondrial membrane potential, attenuated the upregulation of dynamin-related protein 1 (Drp1) and fission 1 (Fis1), and normalized altered mitochondrial DNA (mtDNA) copy number. The mRNA levels of mtDNA-encoded genes (mtND3, mtCO2, and mtcyb) and the nuclear-encoded gene Sdhb were altered. Kidney metabolomic analysis revealed metabolic alterations associated with GY007 supplementation in fluoride-exposed mice, identifying eight significantly altered metabolites. This study provides evidence supporting the development of probiotic interventions to mitigate fluoride-associated renal injury in settings with elevated fluoride concentrations in drinking water.

**Supplementary Information:**

The online version contains supplementary material available at 10.1186/s13568-026-02031-7.

## Introduction

Fluorine (F) is relatively abundant in the Earth’s crust and occurs in natural environments primarily as fluoride (F^−^), typically bound in minerals and other compounds. Environmental accumulation of fluoride can lead to regional contamination and increased human exposure, representing a substantial global public health burden (Su et al. 2021). To mitigate the adverse health effects of excessive fluoride exposure, the World Health Organization (WHO) recommends a guideline value for fluoride in drinking-water of 1.5 mg/L (WHO 2011). However, in endemic areas, groundwater fluoride concentrations can exceed the guideline by an order of magnitude. A compilation of 5464 shallow groundwater samples in China reported fluoride concentrations ranging from 0 to 60 mg/L (Zhao et al. 2022). A review study reported fluoride concentrations in multiple industrial effluents ranging from 250 to 1500 mg/L, with concentrations approaching approximately 10,000 mg/L documented in some cases (Solanki et al. 2022). Moreover, fluoride contamination in food can further increase dietary exposure and broaden the population affected (Li et al. 2023).One study reported total fluoride concentrations in tea of 47.05–291.98 mg/kg (Satou et al. 2021). Zhang et al. (2025) reported that chili peppers roasted using fluoride-rich coal as fuel contained fluoride concentrations up to 869.82 mg/kg. A global review indicates that fluoride contamination in groundwater affects more than 100 countries and over 200 million people worldwide (Shaji et al. 2024). In China, except for Shanghai and Hainan, elevated fluoride levels have been reported across most provinces, placing China among the countries most severely affected by fluoride contamination worldwide (Zhang et al. 2023).

Fluoride can exert adverse effects on living organisms. In addition to dental and skeletal fluorosis, fluoride exposure has been associated with a range of non-skeletal effects, including impaired renal and hepatic function (Malin et al. 2019). Given its central role in fluoride excretion, the kidney is among the organs with the highest fluoride burden (Meenakshi et al. 2025). In healthy adults, approximately 60% of daily fluoride intake is excreted in the urine; in contrast, children excrete a lower proportion, with about 45% of daily fluoride intake eliminated in the urine (Villa et al. 2010; Wu et al. 2021). Among cell types across multiple organs, renal cells appear to be particularly susceptible to fluoride (Dharmaratne 2019; Hongslo et al. 1980; Lavanya et al. 2025). Experimental studies indicate that fluoride can injure both glomerular and tubular structures, characterized histopathologically by tubular epithelial cell damage and inflammatory cell infiltration, leading to impaired renal function and disruption of tubular reabsorption and glomerular filtration (Dharmaratne 2019; Hu et al. 2023; Lavanya et al. 2024; Wu et al. 2021; Zheng et al. 2024). In a cross-sectional study of children, fluoride exposure was associated with higher kidney injury molecule-1 (Kim-1) levels and an abnormal estimated glomerular filtration rate (eGFR; Malin et al. 2019). Another study reported higher fluoride concentrations in drinking water and blood in patients with chronic kidney disease of unknown etiology (CKDu) than in healthy controls (Nanayakkara et al. 2020). Preclinical investigation suggested for the nephrotoxic effect of fluoride exposure, and are linked to upregulated oxidative stressors and initiation of apoptosis/necropsis (Sharma et al. 2023; Tian et al. 2019; Wang et al. 2020a; Zheng et al. 2024). Therefore, effective strategies are needed to prevent or mitigate fluoride-induced nephrotoxicity.

Animal studies suggest that fluoride exposure can disrupt the intestinal microbiota, accompanied by structural changes in the intestine and effects on other organs such as the kidneys and brain (Sun et al. 2020a, 2025; Xin et al. 2020, 2021a). Microbiome research increasingly follows a discovery-to-validation pipeline: microbiome profiling identifies candidate taxa, which are then tested in vivo using defined strains to evaluate effects beyond the gut (Gui et al. 2024; Routy et al. 2018; Yuan et al. 2019; Yang et al. 2025). This strategy has also been adopted in the context of kidney disease (Miao et al. 2024). *Bifidobacterium* spp. are a commonly used probiotic species and have been extensively characterized (Lee et al. 2023; Sanders et al. 2019). Previous studies have reported renoprotective effects of specific *Bifidobacterium* strains in various kidney disease models, suggesting their therapeutic potential (Wang et al. 2020b; Mao et al. 2025; Meng et al. 2024). However, probiotic effects are often strain- and disease-specific; therefore, validation at the strain level is critical (McFarland et al. 2018; Su et al. 2020).

First, we established a mouse model of prolonged high-fluoride exposure and employed metagenomic sequencing to identify bacterial species associated with renal function. We then evaluated the protective effects of *Bifidobacterium animalis* GY007 in a short-term high-fluoride exposure mouse model by measuring renal injury biomarkers, antioxidant capacity, inflammatory cytokine levels, mitochondrial homeostasis, and renal histopathology. Finally, we performed an analysis of mouse kidney samples through untargeted metabolomics.

## Materials and methods

### Animals

These procedures were performed as previously described (Xin et al. 2023). Male C57BL/6 J mice (3-week-old) were used and given ad libitum access to standard chow and water. All animal care and experimental procedures were performed in accordance with protocols approved by the Sichuan Agricultural University Animal Ethical and Welfare Committee (approval number: 20250405; experimental animal license number: SYXKchuan2024-0187).

### Exposure rationale and experimental design

*Exposure rationale*: This study used high-fluoride exposure mouse models. The term ‘high-fluoride exposure’ refers to a situation where the fluoride concentration in water exceeds 1.5 mg/L according to the WHO standards (WHO 2011). In Experiment 1, long-term exposure to sodium fluoride (25/50 ppm) in drinking water for 56 weeks was conducted. The fluoride doses and experimental duration were as described in previous studies (Xin et al. 2023, 2025). According to the dose conversion formula of the U.S. Food and Drug Administration (FDA), these quantities are approximately 2.03 ppm and 4.05 ppm, equivalent to human doses (Supplementary Material). Documented adult fluoride intakes from drinking water range from 0.84 to 27.1 mg/day (Fawell et al. 2006), corresponding to an equivalent drinking-water concentration of 0.19 to 6.02 ppm (1 ppm ≈ 1 mg/L), assuming a 55 kg adult consumes 4.5 L/day (Xin et al. 2021b). In Experiment 2, a short-term fluoride exposure model (24 mg/kg/day sodium fluoride by gavage for 8 weeks) was used. The fluoride doses and experimental duration were as described in previous studies (Huang et al. 2024; Sun et al. 2025; Xin et al. 2025). For an adult weighing 55 kg and with a daily water intake of 4.5 L, the equivalent human dose is approximately 23.8 ppm (Supplementary Material).

*Experimental design*: First, all mice underwent a 1-week acclimation period. Throughout the study, all mice were maintained under identical husbandry conditions and were given the same standard chow and drinking water. Except for the fluoride exposure treatment in the study design, no additional fluoride exposure interventions will be applied.

Experiment 1: The workflow was carried out according to our previously published protocols (Xin et al. 2023, 2025). Four-week-old mice were provided ad libitum access to drinking water supplemented with sodium fluoride (25 ppm, F-Low or 50 ppm, F-High) up to 60 weeks of age. The control mice received drinking water without fluoride. After 56 weeks of exposure, urine and serum samples were collected. Mice (n = 10 per group) were euthanized humanely, and the kidneys and ileum were quickly dissected. For histological analysis, left kidneys from three mice per group were fixed in 4% paraformaldehyde. Contents of the ileum were collected for metagenomic sequencing. Experiment 1 aimed to investigate renal phenotypes and microbial characteristics following long-term fluoride exposure.

Experiment 2: The metagenomic analysis from Experiment 1 identified *Bifidobacterium animalis* (*B. animalis*) as a candidate species potentially linked to fluoride-induced renal injury. A subsequent experiment was conducted to validate the effects of *B. animalis subsp. animalis* GY007 on fluoride-induced renal injury. The grouping and processing procedures are shown in Fig. 3A. All groups received control drinking water throughout the study. The F + Bi group was gavaged daily with 0.2 mL of sodium fluoride solution (24 mg/kg) in sterile normal saline, then given 0.2 mL of GY007 suspension in sterile normal saline. The F group received 0.2 mL sodium fluoride solution and 0.2 mL normal saline, while the Ctrl group received only saline. The treatments were administered for 8 weeks. After 8 weeks of treatment, mice were weighed, and urine, serum, and kidney samples were collected as described in Experiment 1 (n = 12 per group). For histological examination, left kidneys from three mice per group were fixed in 4% paraformaldehyde. For TEM, renal tissues were fixed in 2.5% glutaraldehyde. Experiment 2 was designed to determine whether the strain *B. animalis* GY007 could alleviate fluoride-induced kidney damage.

### Metagenomic and bioinformatics analysis

A total of thirty metagenomic profiles were generated from intestinal contents (n = 10 per group). Detailed procedures are provided in the Supplementary Material.

### Bacterial strain and culture

*B. animalis subsp. animalis* GY007 (CCTCC AB 2025172) was cultured in de Man, Rogosa and Sharpe (MRS) medium at 37 °C for 24 h to ensure stable growth and metabolic activity. Bacterial pellets were collected by centrifugation of the third-passage culture at 4000 × g for 15 min. The precipitates were washed three times with sterile physiological saline. The concentration of GY007 was adjusted to 1 × 10^9^ CFU mL^−1^ using the colony counting method for in vivo experiments.

### Biochemical detection

Mice serum and urine samples were centrifuged, and the supernatants were used for subsequent experiments. The supernatants obtained from grinding the kidneys were used for the measurement of Drp1 and Fis1 level. A total of 24 paired serum and urine samples were randomly selected for analysis (n = 8 per group). Kit information is provided in the Supplementary Material.

To assess mitochondrial membrane potential (MMP), mitochondria were isolated from 150 mg of fresh kidney tissue using a commercial mitochondria isolation kit (SM0020; Solarbio, Beijing, China). The isolated mitochondria were resuspended in the kit-provided storage buffer and kept on ice for immediate use. Mitochondrial protein concentration was determined using a BCA Protein Assay Kit (Boster, Wuhan, China) to normalize the amount of mitochondrial protein used for subsequent MMP measurements. MMP in freshly isolated mitochondria was measured using a JC-1-based mitochondrial membrane potential assay kit (M8650; Solarbio, Beijing, China) according to the manufacturer’s instructions. Briefly, JC-1 is a MMP-sensitive probe that forms polymer at high MMP and remains in the monomer form when MMP is low. Changes in MMP were assessed based on fluorescence intensity. The isolated mitochondria were diluted in 0.9% normal saline to a final protein concentration of 200–400 µg/mL and then incubated with the JC-1 probe at 37 °C for 20 min. Fluorescence intensity was measured following a microplate method using a VarioSkan LUX multimode reader (Thermo Scientific). The JC-1 polymer fluorescence was measured at 525/590 nm (excitation/emission wavelengths), and the JC-1 monomer fluorescence was measured at 490/530 nm (excitation/emission wavelengths). The MMP value was represented by ΔΨm (polymer fluorescence/monomer fluorescence).

### Metabolite detection and bioinformatics analysis

In this study, metabolomic profiles were generated from 36 kidney samples (n = 12 per group). Metabolite detection and bioinformatics analysis were performed as previously described (Gan et al. 2025).

### Real-time quantitative PCR (RT-qPCR) in kidney tissue

A total of 15 kidney samples were randomly selected for analysis (n = 5 per group). RNA extraction, DNA extraction, RT-qPCR, and the calculation of relative target mRNA expression levels were performed as previously described (Xin et al. 2023). The mtDNA copy number was determined by qPCR using primers specific for mtND1 (mitochondrial gene) and the 18S rRNA gene (nuclear reference gene). The expression levels of the other target genes were normalized to β-actin as an internal control. Primer sequences are detailed in Supplementary Table S1.

### Histology

For TEM, kidney tissues were rinsed in 0.1 M phosphate buffer (PB; pH 7.4) and post-fixed in 1% osmium tetroxide (OsO₄) in 0.1 M PB (pH 7.4) for 2 h at room temperature (20–25 °C). The samples were then washed in 0.1 M PB (pH 7.4), dehydrated through ethanol, embedded in EMBed 812, and polymerized at 65 °C for at least 48 h. Ultrathin Sects. (60–80 nm) were cut on an ultra-microtome, double-stained with 2% uranium acetate saturated alcohol solution and 2.6% lead citrate, and observed under TEM (HT7800/HT7700, HITACHI, Japan) at an accelerating voltage of 80 kV. Images were captured.

For Hematoxylin and eosin (HE), Masson’s trichrome (Masson), and periodic acid-Schiff (PAS) staining, kidneys were processed, paraffin-embedded, and sectioned after fixation in paraformaldehyde. Sections were stained with HE, Masson, and PAS according to previously published methods (Deng et al. 2024; Xin et al. 2021b; Xu et al. 2024). Images were captured using an OLYMPUS microscope.

Single-label immunofluorescence staining was performed as previously described (Sun et al. 2025). Sections were incubated overnight at 4 °C with one of the following primary antibodies diluted in PBS: rabbit monoclonal anti-TNF-α (1:50), anti-IL-6 (1:200), anti-IL-10 (1:200), or anti-4-hydroxynonenal (anti-4-HNE; 1:100). Detection and collection of images using fluorescent microscopy: DAPI glows blue under UV excitation at 330–380 nm with an emission wavelength of 420 nm; and CY3 glows red under excitation at 510–560 nm with an emission wavelength of 590 nm.

### Statistical analysis

Normal distributions were assessed using the Shapiro–Wilk normality test. If the data were not normally distributed, normality tests were repeated after log-transformation. Normally distributed data with homogeneity of variance were analyzed using t-tests (two independent samples) or one-way ANOVA with LSD post hoc multiple analysis (comparisons among multiple groups). Normally distributed data with heterogeneity of variance among multiple groups were analyzed using one-way ANOVA with Dunnett’s T3 post hoc analysis. Data that did not reach a normal distribution were tested using the Kruskal–Wallis test at *P* < 0.05, followed by the Wilcoxon rank-sum test. Linear trend across fluoride concentrations (0/25/50 ppm) was assessed by linear regression with dose coded as an ordered numeric variable (0, 1, 2; per 25 ppm). Effect sizes were reported as the regression coefficient (change per 25 ppm) with 95% confidence intervals. For outcomes that remained non-normally distributed after log-transformation, a non-parametric trend test was performed using Spearman’s rank correlation between dose order and outcome (with bootstrap 95% CI). Data are presented as the mean ± standard deviation and were analyzed using IBM SPSS Statistics 27. *P < 0.05, * *P < 0.01, * **P < 0.001 were considered significant. The mapping software used was GraphPad Software 8.0.

## Results

### Chronic fluoride exposure is associated with renal injury in mice

**Fig. 1:**
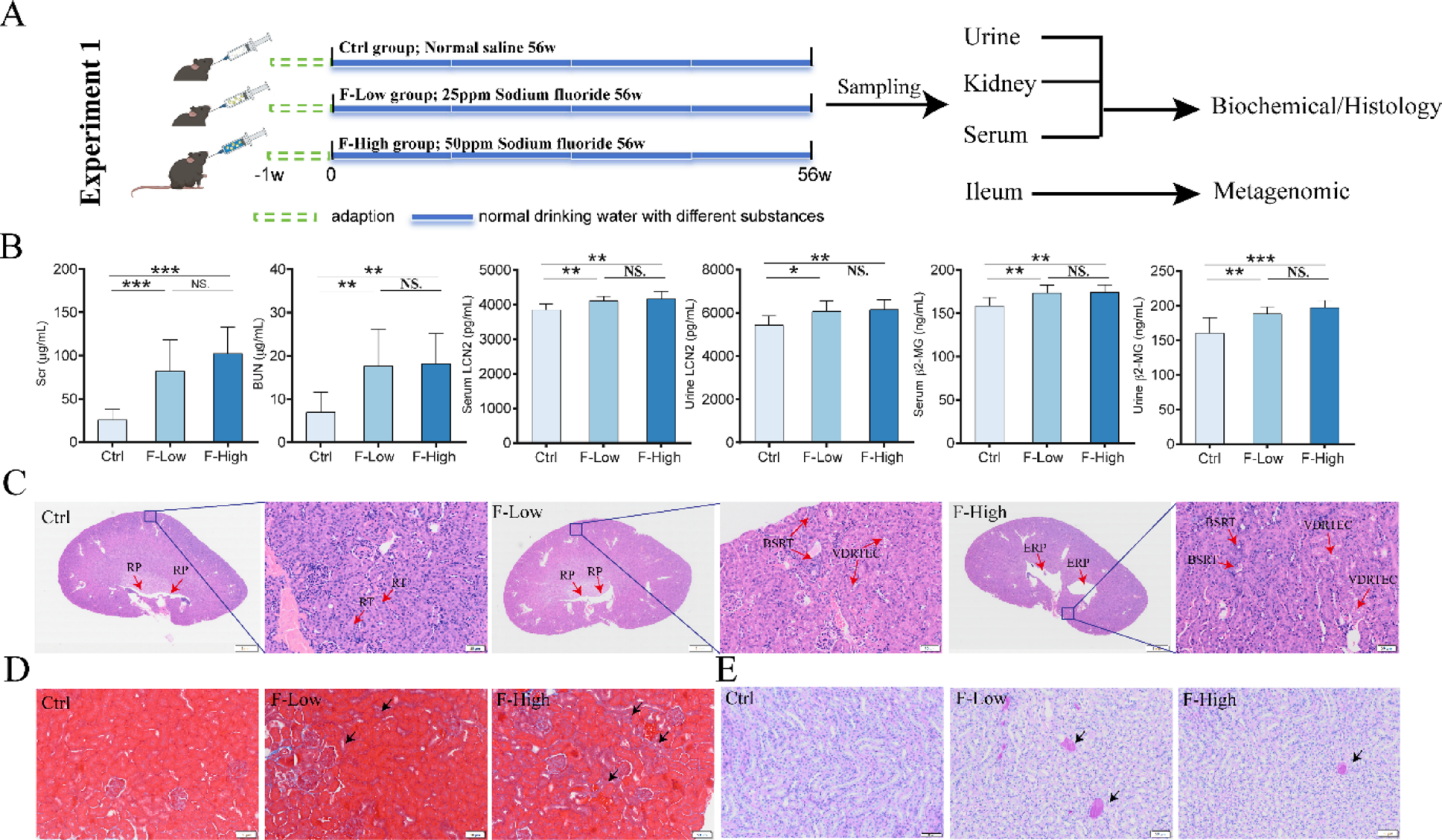
Chronic fluoride exposure resulted in renal damage in mice. **A** Diagram illustrating the design of Experiment 1. **B** Content of Scr, BUN, LCN2, β2-MG of serum or urine (n = 8). **C**–**E** Representative photographs of HE (The left side of each group: 0.6×, 1 mm; The right side of each group: 10×, 50 μm), Masson (10×, 50 μm) and PAS (10×, 50 μm). RP, Renal Palvis; ERP, Expanded Renal Palvis; RT, Renal tubules; BSRT, Basophilic substances within the renal tubules; VDRTEC, Vacuolar degeneration of renal tubular epithelial cells

Using the animal model described previously, we investigated the effects of sustained fluoride exposure on renal function (Fig. 1A). The levels of serum creatinine (Scr), serum urea nitrogen (BUN), as well as serum and urinary levels of LCN2 and β2-MG, were significantly higher in both fluoride-treated groups than in the Ctrl group (*P* < 0.01; Fig. 1B). Kidney pathology was evaluated using HE, Masson, and PAS staining. Representative images are shown in Fig. 1C–E. Light microscopy of HE-stained kidney sections showed intact tubular architecture and a normal renal pelvis in control mice. Histological examination revealed basophilic intratubular material and vacuolar degeneration of renal tubular epithelial cells in the F-Low and F-High groups. Additionally, renal pelvic dilatation was observed in the F-High group. Masson staining showed that both fluoride-exposed groups (F-Low and F-High) exhibited tubular injury and increased blue-stained collagen deposition. PAS staining showed intraluminal deposits in the renal collecting ducts in both fluoride-exposed groups (F-Low and F-High). Taken together, chronic fluoride exposure (56 weeks) in mice was associated with renal functional impairment and kidney lesions. Notably, the severity of kidney injury did not differ significantly between the F-Low and F-High groups. Futhermore, linear regression treating dose as an ordered numeric variable (0, 1, 2; per 25 ppm) showed a significant dose-related increase for Scr, BUN, as well as in serum and urinary levels of LCN2 and β2-MG (Extended Data Table 1).

### Inverse association between *B. animalis* abundance and renal injury markers under prolonged fluoride exposure

**Fig. 2:**
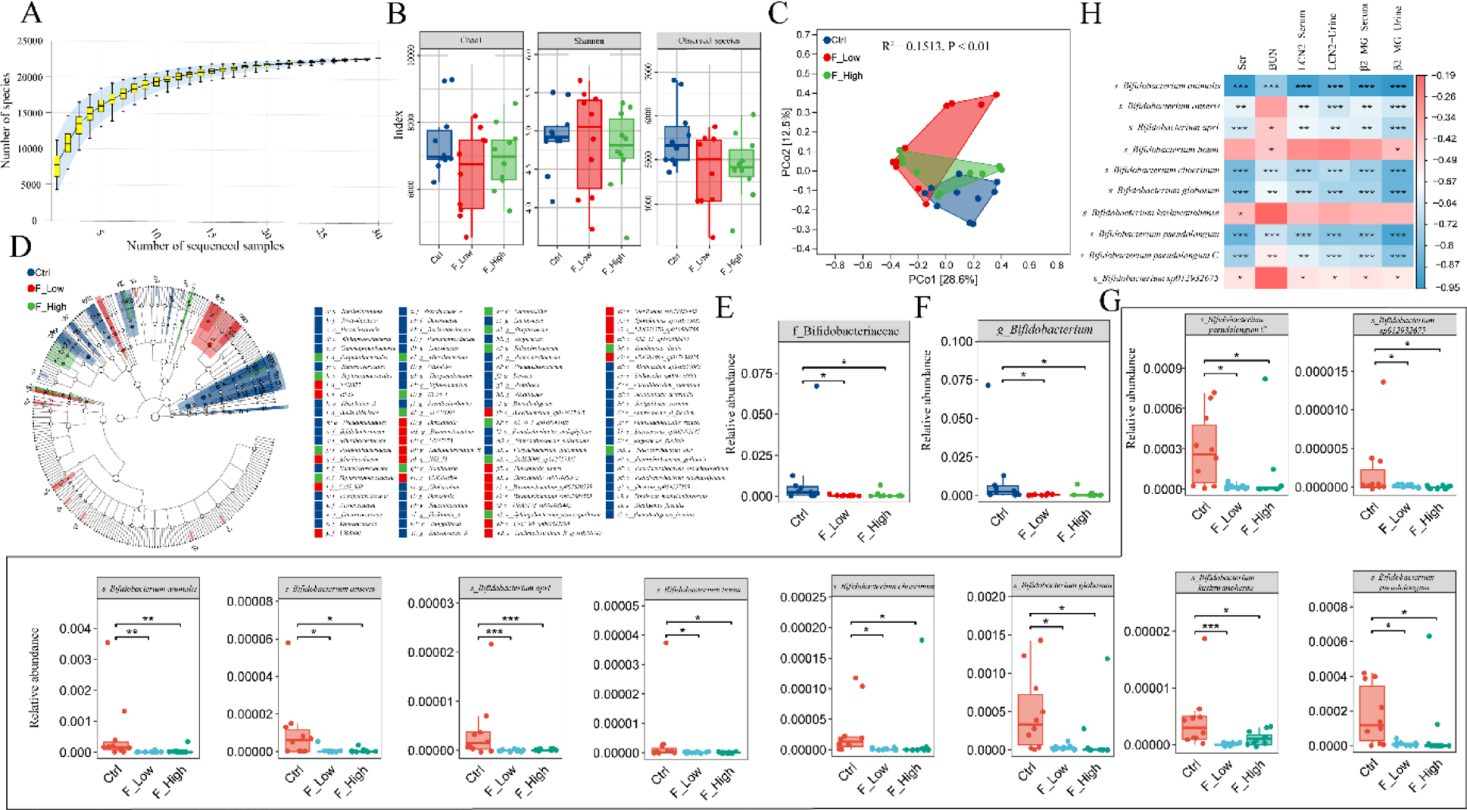
Prolonged fluoride exposure alters ileal microbiota and is associated with reduced *B. animalis* abundance and renal injury markers. **A** Species accumulation curves. The horizontal coordinate is the sample size, and the vertical coordinate is the number of feature species. **B** α-diversity (Chao1, Shannon and Observed species) in mice. **C** PCoA based on Bray–curtis in three groups mice. **D** LEfSe cladogram showing taxa differentially enriched among groups (LDA effect size analysis). **E**, **F** The relative abundances of f_bifidobacteriaceae, *g_Bifidobacterium* in controls and two groups of mice exposed to different fluoride concentrations. **G** In *g_Bifidobacterium*, the relative abundances of 10 species showed significant differences when compared to the Ctrl. **H** Spearman’s rank correlation

The gut microbiota in prolonged fluoride-exposed mice was characterized using metagenomic sequencing. As shown in Fig. 2A, the species accumulation curve approached a plateau, indicating that additional samples contributed little to overall species richness. Therefore, the metagenomic sequencing data generated in this study were sufficient to characterize community diversity and support downstream analyses. As shown in Fig. 2B, the Chao1 index, Shannon index, and observed species richness tended to be lower in both fluoride-treated groups than in the control group; however, these differences were not statistically significant among the three groups. Long-term fluoride exposure significantly altered the β-diversity of the ileal microbiota in mice (Fig. 2C; R^2^ = 0.1513,* P* < 0.01). LEfSe cladogram analysis revealed distinct alterations in the ileal microbiota of aged mice following long-term fluoride exposure (Fig. 2D). Our results suggest that long-term fluoride exposure alters the gut microbial community in mice. In our previous study evaluating probiotics for fluoride-induced renal injury, 16S rRNA gene sequencing showed an altered relative abundance of *Bifidobacterium* in the probiotic intervention group (Xin et al. 2021a). Nevertheless, the taxa could not be resolved at the species level. Therefore, we focused on changes in *Bifidobacterium*. Metagenomic analysis showed no statistically significant differences between groups in the relative abundance of taxa to which *Bifidobacterium* belongs at the phylum, class, or order levels (Actinobacteriota, Actinomycetia, and Actinomycetales; Fig. S1). However, as shown in Fig. 2E, F, the relative abundances of Bifidobacteriaceae and *Bifidobacterium* were significantly reduced in both fluoride-treated groups compared with controls (*P* < 0.05).

Among the 68 annotated *Bifidobacterium* species, the relative abundances of 10 species were significantly reduced in both fluoride-treated groups compared with controls (*P* < 0.05; Fig. 2G; Extended Data Table 2). Further correlation analysis revealed that 10 *Bifidobacterium* species were positively or negatively correlated with renal injury markers (*P* < 0.05; Fig. 2H). The relative abundance of *Bifidobacterium animalis* was negatively correlated with all measured indices of renal impairment and showed the strongest association among the 10 species (*P* < 0.001; Fig. 2H; Extended Data Table 3).

### The effects of *B. animalis subsp. animalis* GY007 on body mass and kidney ratios in mice following exposure of fluoride

**Fig. 3:**
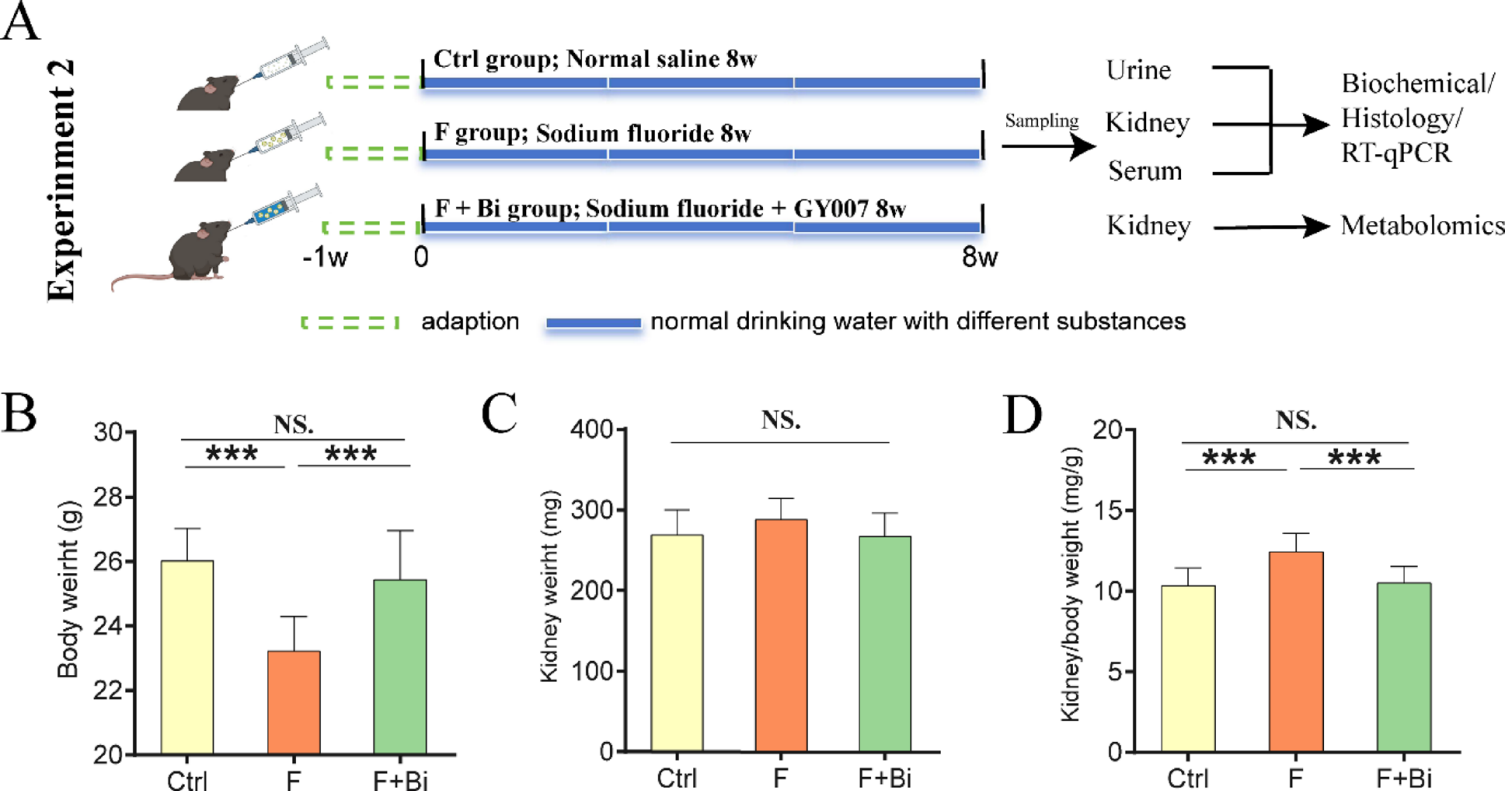
Body and kidney weight, and kidney-to-body weight ratio in mice following GY007 treatment. **A** Schematic of Experiment 2 design. **B** Body weight. **C** Bilateral kidney weight. **D** Kidney-to-body ratio. n = 12

The relative abundance of *Bifidobacterium animalis* in the ileum was significantly negatively correlated with all assessed indices of renal function impairment. Based on these findings, we performed Experiment 2 (Fig. 3A). GY007 significantly increased body weight in mice exposed to fluoride (*P* < 0.001, Fig. 3B). Bilateral kidney weight did not differ significantly among the three groups (Fig. 3C). In contrast, GY007 significantly reduced the kidney-to-body weight ratio compared with fluoride exposure (*P* < 0.001, Fig. 3D).

### Improvement effect of GY007 on the pathology of kidney

**Fig. 4:**
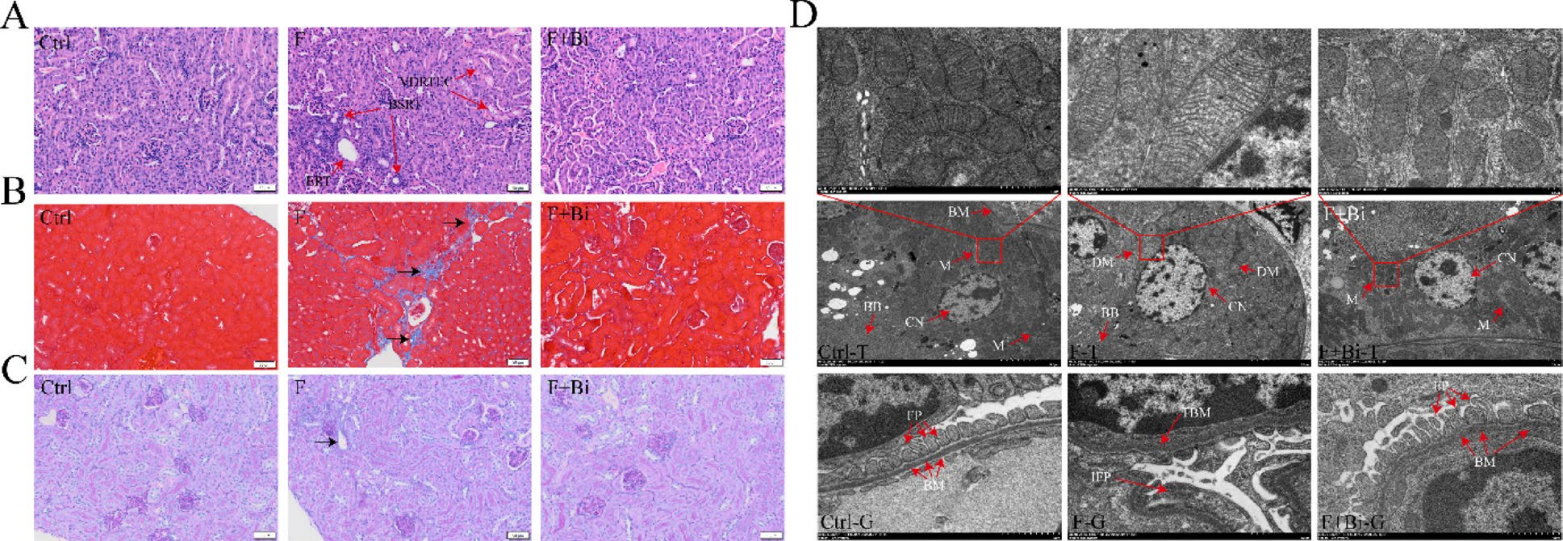
The impact of GY007 on the renal structure following by fluoride. **A**–**C** Representative photographs of HE (10×, 50 μm), Masson (10×, 50 μm) and PAS (10×, 50 μm). BSRT, Basophilic substances within the renal tubules; VDRTEC, Vacuolar degeneration of renal tubular epithelial cells; ERT, Expanded renal tubules. **D** Representative photographs of TEM. M, mitochondrion; DM, damaged mitochondrion; CN, cell nucleus; BM, basement membrane; TBM, Thickened basement membrane; BB, brush border; FP, foot process; IFP, integrated foot process. Ctrl-T: The ultrastructural morphology of the renal tubular region in the control group; Ctrl-G: The ultrastructural features of the renal glomerular area in the control group; The remaining annotations are of an analogical nature. The information on observation multiples and scales is as follows. Ctrl-T, F-T, and F + Bi-T: 30 K × , 1 μm (above); 6 K × , 5 μm (below). Ctrl-G, F-G, and F + Bi-G: 30 K × , 1 μm

To evaluate the protective effects of GY007 against renal pathological changes, we assessed kidney sections by HE, Masson, and PAS staining, as well as TEM. HE staining revealed that lesions in fluoride-treated kidneys were predominantly localized to the renal tubules, characterized by basophilic material deposition, epithelial cell vacuolation, and tubular dilatation (Fig. 4A). Masson staining showed obvious deposition of collagen fibrosis in the kidneys following fluoride exposure (Fig. 4B). PAS staining showed renal tubular degeneration after fluoride exposure (Fig. 4C). These findings were consistent with those observed in Experiment 1. The use of GY007 significantly attenuated fluoride-induced renal injury. Renal organelle dysfunction can be induced by multiple stressors that compromise cellular function and survival, ultimately exacerbating renal pathology (Hu et al. 2024; Song et al. 2023). Therefore, restoring or maintaining organelle function represents a promising therapeutic strategy for the treatment of renal diseases. Kidney tissues from the three groups were examined by TEM. Compared with control mice, fluoride exposure induced mitochondrial damage in renal tissue, as evidenced by reduced or absent cristae and mitochondrial swelling (DM). Compared with the Ctrl group, the F group exhibited glomerular basement membrane thickening (TBM) and foot process effacement (IFP). After treatment with GY007, BM thickening, FP integrity, and mitochondrial morphology were restored (Fig. 4D). These results indicated that GY007 alleviates fluoride-induced pathological kidney injury in mice.

### GY007 alleviates kidney function impairment and oxidative damage induced by fluoride exposure

**Fig. 5:**
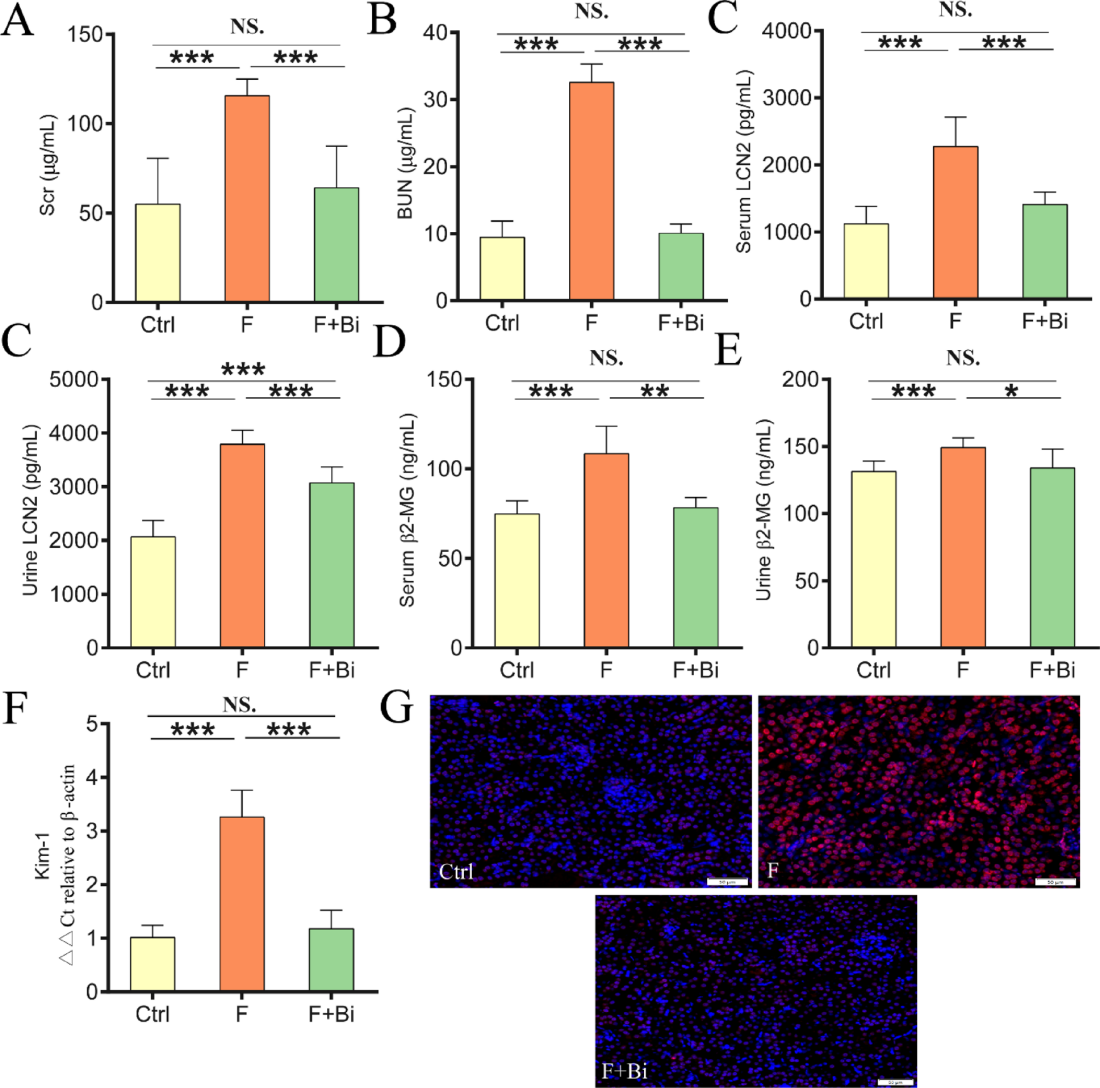
GY007 has the potential to mitigate the decline in renal function induced by fluoride. **A**–**E** Content of Scr, BUN, LCN2, β2-MG of serum or urine (n = 8). **F** The mRNA expression levels (n = 5) of Kim-1. **G** Representative immunofluorescence image of 4-HNE (20×, 50 μm). The proteins expression of 4-HNE are stained red

**Fig. 6:**
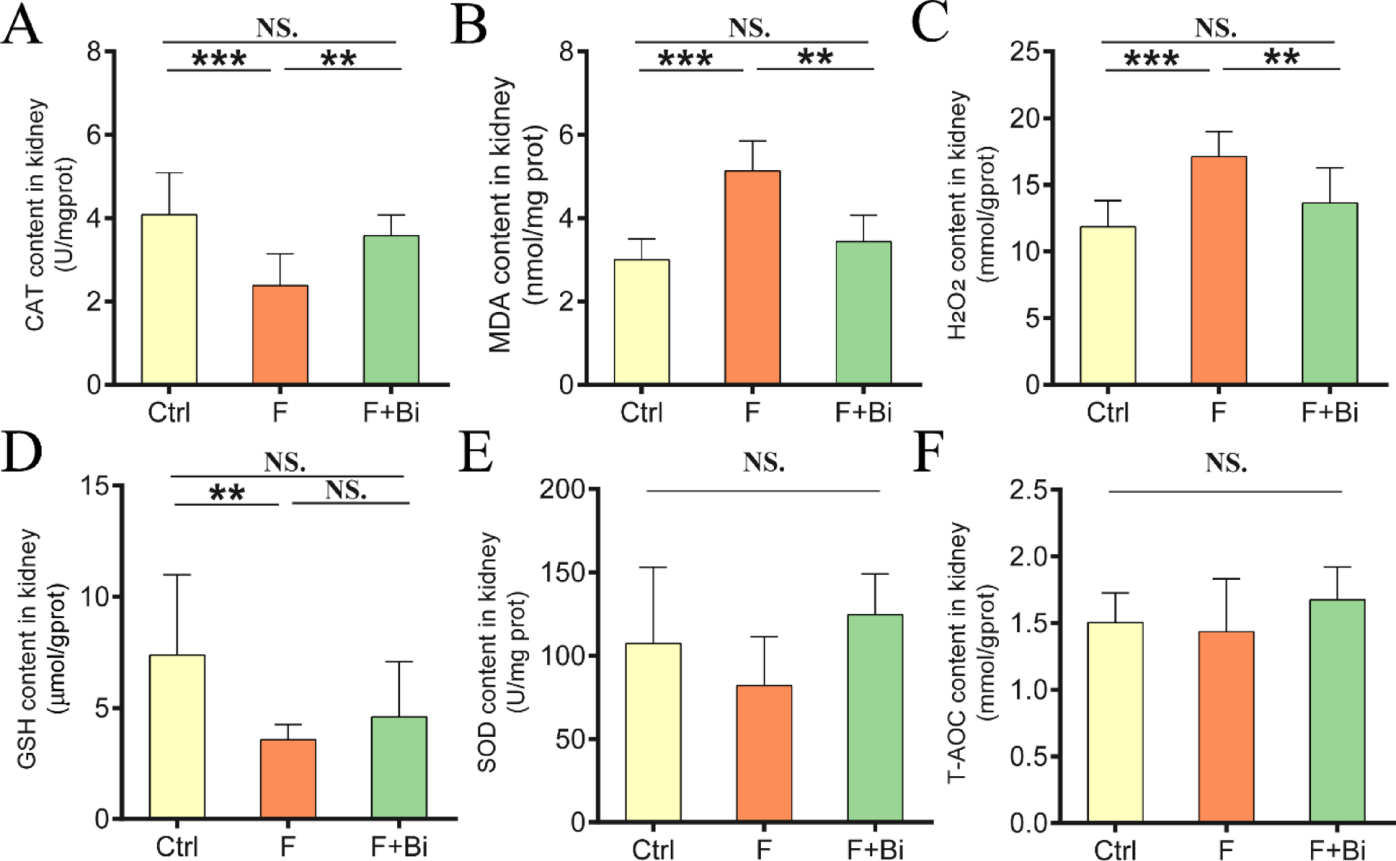
Kidney antioxidant response to elevated fluoride and GY007. **A** Activities or contents (n = 8) of CAT. **B** Activities or contents (n = 8) of MDA. **C** Activities or contents (n = 8) of H_2_O_2_. **D** Activities or contents (n = 8) of GSH. **E** Activities or contents (n = 8) of SOD. **F** Activities or contents (n = 8) of T-AOC

To evaluate the effects of GY007 on renal function, Scr, BUN, serum and urinary levels of β2-MG and LCN2 were measured. Consistent with Experiment 1, fluoride exposure significantly increased concentrations of renal dysfunction biomarkers (*P* < 0.001; Fig. 5A–E). GY007 treatment significantly reduced these biomarker levels. The mRNA expression of Kim-1 was increased (*P* < 0.001) after fluoride exposure, and GY007 reduced this increase (*P* < 0.001, Fig. 5F). Immunofluorescence experiments revealed enhanced renal lipid peroxidation in the fluoride treatment group. GY007 reduced lipid peroxidation in the kidneys of the mice (Fig. 5G). We further assessed renal oxidative stress. Significant alterations (*P* < 0.001) in catalase (CAT) activity, malondialdehyde (MDA) and hydrogen peroxide (H_2_O_2_) levels were observed after fluoride exposure (Fig. 6A–C). GY007 significantly reversed these trends (*P* < 0.01, Fig. 6A–C). Fluoride reduced the renal reduced glutathione (GSH) content in mice (*P* < 0.01), and GY007 showed a trend toward restoring GSH levels, but this was not statistically significant (Fig. 6D). Fluoride and GY007 did not significantly affect superoxide dismutase (SOD) activity or total antioxidant capacity (T-AOC) in kidney tissue of mice (Fig. 6E, F). These results suggested that *B. animalis subsp. animalis* GY007 improved renal function and alleviates oxidative stress in fluoride-exposed mice.

### GY007 alleviates the fluoride-induced renal inflammation and improves mitochondrial homeostasis

**Fig. 7:**
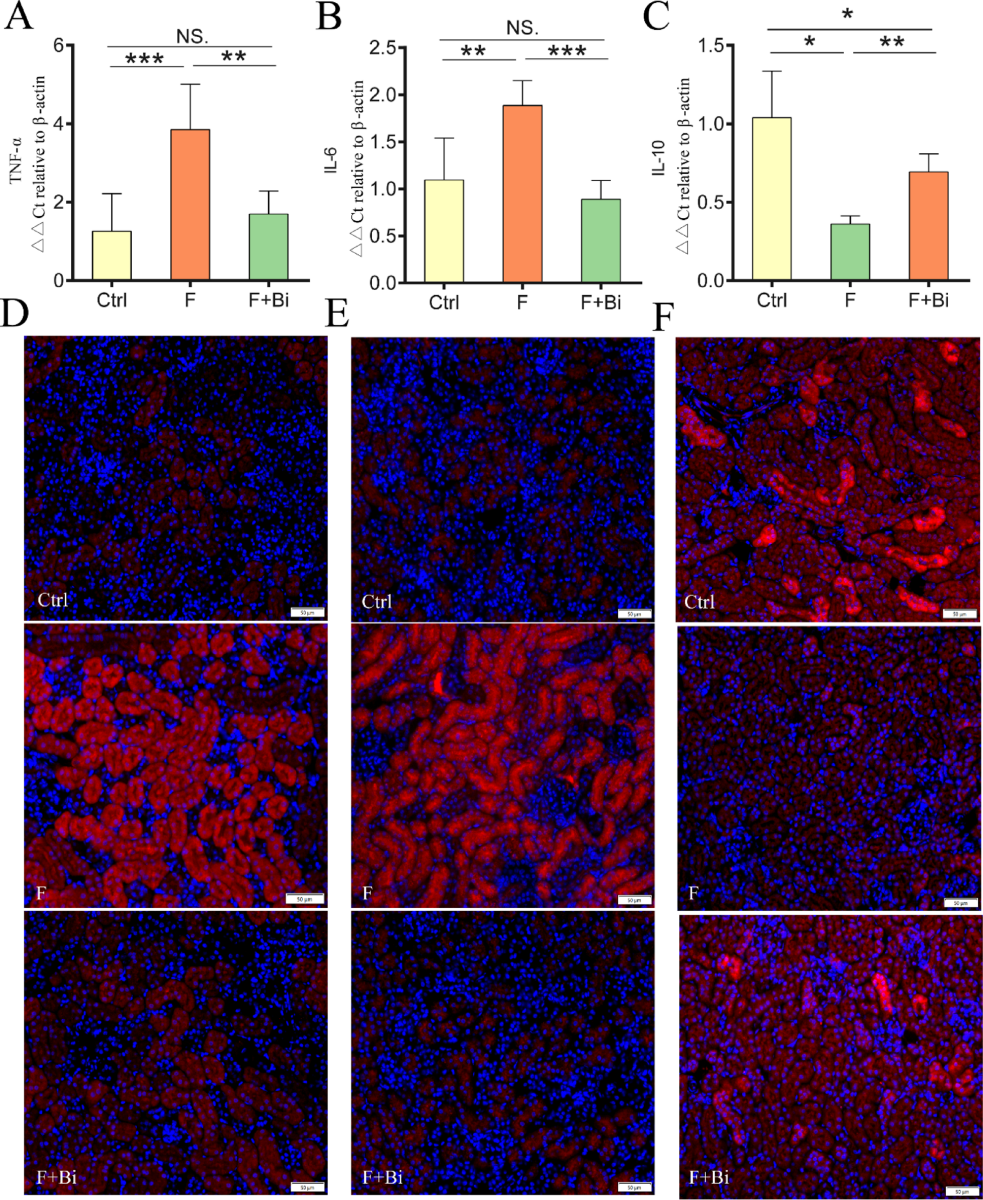
Assessment of renal inflammatory factor levels. **A** The levels (n = 5) of TNF-α. **B** The levels (n = 5) of IL-6. **C** The levels (n = 5) of IL-10. **D**–**F** Representative immunofluorescence image of them, arrange in order (20×, 50 μm). The proteins are stained red

**Fig. 8:**
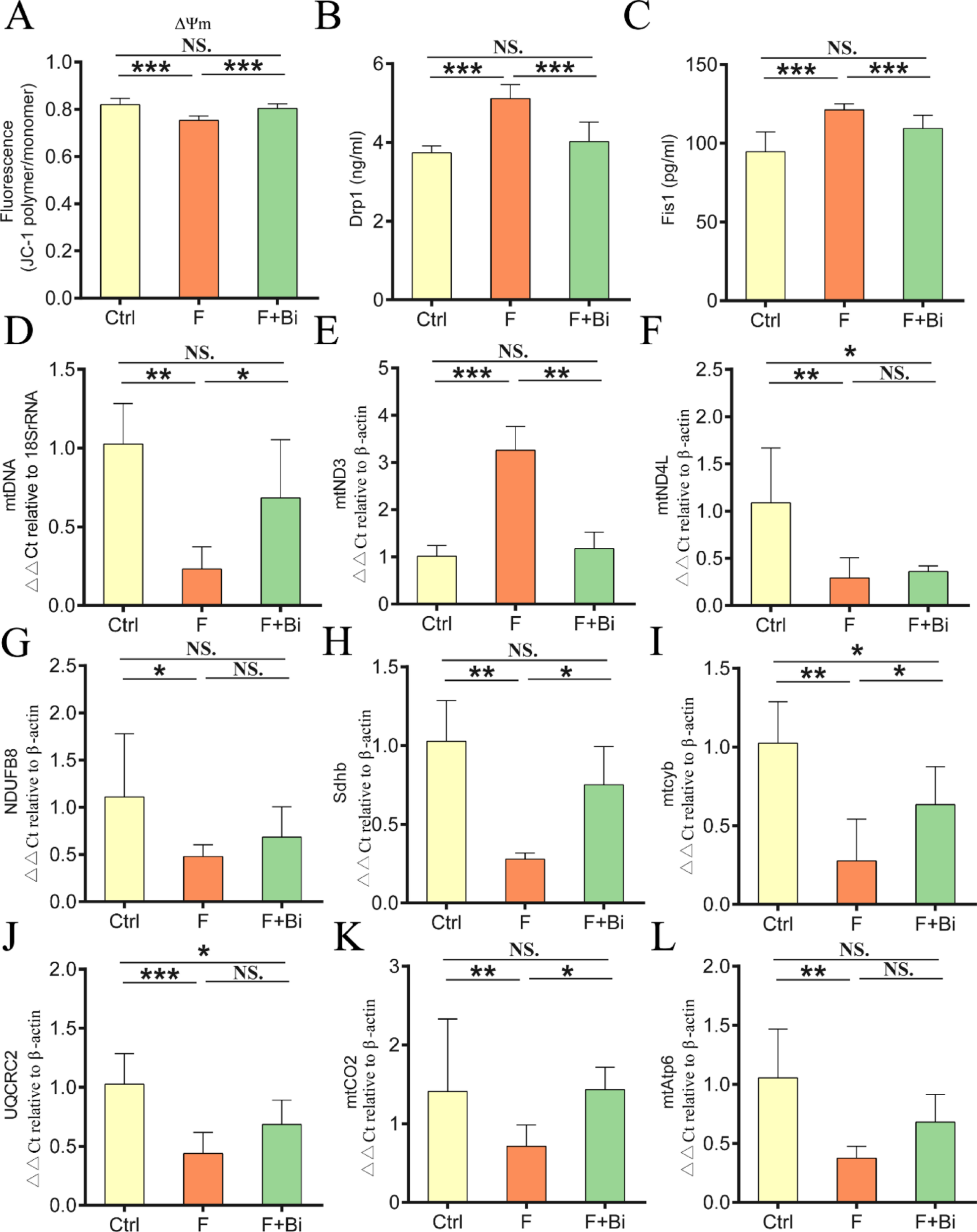
GY007 improves mitochondrial homeostasis in kidney of mice. **A** Renal MMP. n = 8. **B**, **C** The levels of Drp1 and Fis1. n = 8. **D** Renal mtDNA content. n = 5. **E–L** mRNA levels of kidney subunits of the mitochondrial electron transport complexes. n = 5

Fluoride significantly altered renal inflammatory cytokine levels, including TNF-α, IL-10, and IL-6 (*P* < 0.05; Fig. 7A–C). GY007 treatment significantly reversed this changes (*P* < 0.05). Immunofluorescence analyses showed results consistent with the mRNA expression levels (Fig. 7D–F). TEM revealed mitochondrial ultrastructural abnormalities in renal tissue. To further assess mitochondrial damage, we conducted a measurement of MMP in mice. As shown in Fig. 8A, the mitochondrial membrane potential in the fluoride-exposed group was significantly decreased compared to the control group (*P* < 0.01). In light of the observed mitochondrial structural damage, we assessed the levels of mitochondrial fission proteins in the kidney mitochondria of the experimental mice. As shown in Fig. 8B, C, the protein levels of Drp1 and Fis1 in the fluoride-exposed group were significantly increased (*P* < 0.01). Treatment with GY007 significantly restored mitochondrial membrane potential and normalized fluoride-induced increases in mitochondrial fission protein levels (*P* < 0.01). To investigate how fluoride affects the molecular regulation of mitochondria, we examined respiratory chain complex subunits in kidney mitochondria. After exposure to fluoride, the relative content of kidney mtDNA was decreased (*P* < 0.01), suggesting a disruption in mitochondrial biogenesis (Fig. 8D). The mRNA expression levels of mtDNA-encoded genes (mtND3, mtND4L, mtcyb, mtCO2, and mtAtp6) and nuclear DNA-encoded genes (Sdhb, NDUFB8, and UQCRC2) were significantly changed (*P* < 0.05; Fig. 8E–L). GY007 treatment significantly restored the mRNA levels of mtDNA, mtND3, Sdhb, and mtCO2 (*P* < 0.05), restoring them to near-control levels. GY007 significantly increased the mRNA levels of mtcyb (*P* < 0.05); however, the levels remained significantly lower than the Ctrl. Furthermore, mRNA levels of several respiratory chain-related subunits (mtND4L, NDUFB8, UQCRC2, and mtAtp6) were numerically higher in the F + Bi group, but the differences were not statistically significant.

### GY007 supplementation affected the renal metabolome

**Fig. 9:**
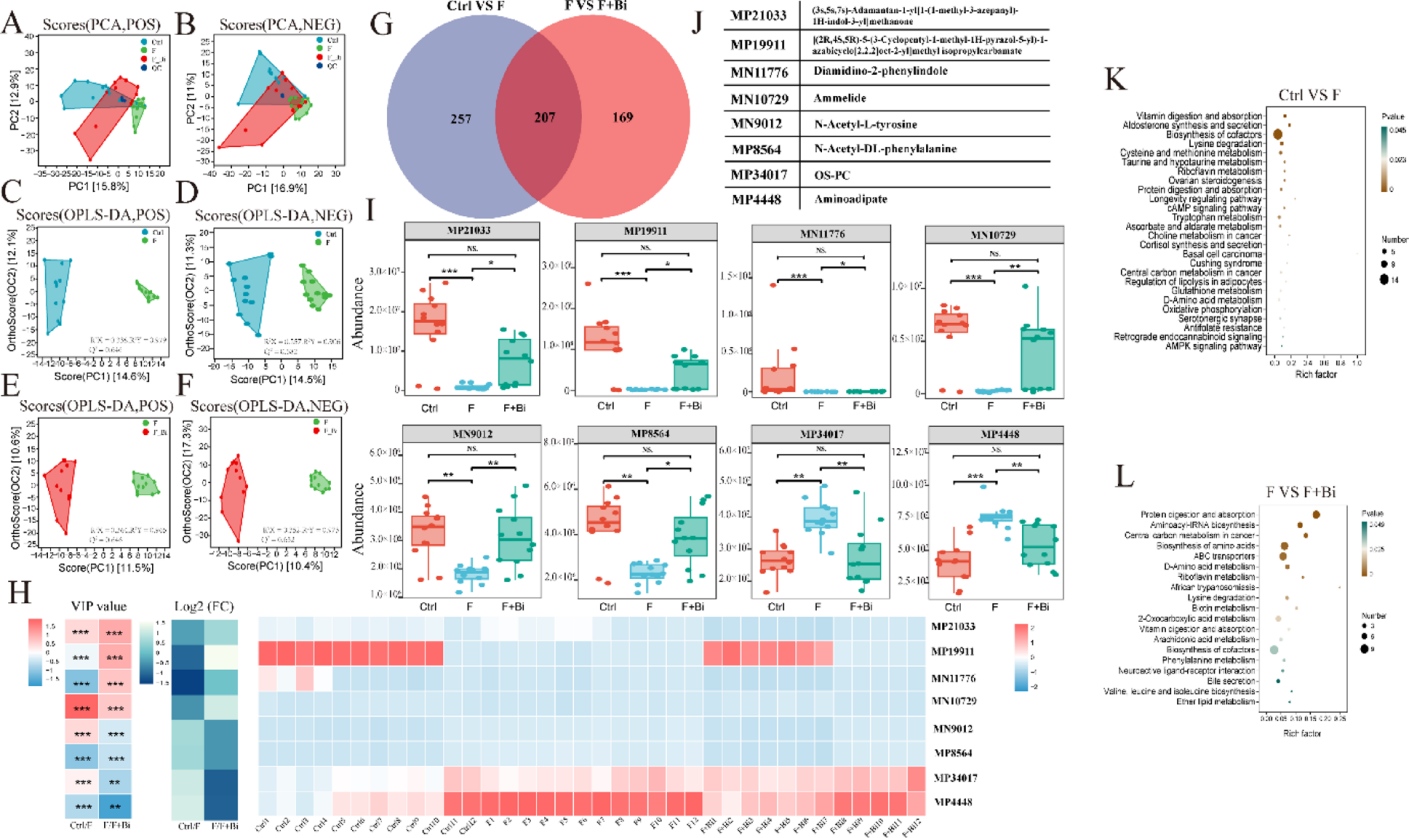
The impact of GY007 on kidney metabolites with high flouride exposure. **A**, **B** PCA plots. **C**–**F** OPLS-DA plots of Ctrl VS F and F VS F + Bi. **G** Venn diagram. **H** Expression analysis and VIP values. **I** Box plot of significant differences in metabolites. **J** The names of the differentially expressed metabolites in Fig. H-I. **K**, **L** KEGG enrichment pathways of differentially expressed metabolomics between Ctrl VS F and F VS F + Bi

In total, 1607 metabolites were identified (positive ions: 803; negative ions: 804). PCA score plots showed a separation of samples among the groups, indicating distinct metabolic profiles (Fig. 9A, B). Further OPLS-DA analysis indicated good model performance, as reflected by the Q^2^ values for Ctrl vs F (positive ion mode: 0.646; negative ion mode: 0.582) and F vs F + Bi (positive ion mode: 0.646; negative ion mode: 0.632; Fig. 9C–F). OPLS-DA score plots showed clear separation among the groups. Compared with control mice, group F showed 464 significantly altered metabolites, including 234 increased and 230 decreased metabolites (Fig. 9G; Extended Data Table 4). Compared with the F group, 376 metabolites were significantly altered in the F + Bi group, including 200 increased and 176 decreased metabolites (Fig. 9G; Extended Data Table 5). In addition, 207 differential metabolites were common to both comparisons (Fig. 9G). Among these, eight metabolites had VIP > 2.0 (Fig. 9H–J; Extended Data Tables 4–6): MP21033 [(3 s,5 s,7 s)-Adamantan-1-yl[1-(1-methyl-3-azepanyD)-1H-indol-3-yl]methanone], MP19911 [[(2R,4S,5R)-5-(3-Cyclopentyl-1-methyl-1H-pyrazol-5-yl)-1-azabicyclo[2.2.2]oct-2-yl]methylisopropylcarbamate], MN11776 [Diamidino-2-phenylindole], MN10729 [Ammelide], MN9012 [N-Acetyl-L-tyrosine], MP8564 [N-Acety-DL-phenylalanine], MP34017 [OS-PC], and MP4448 [Aminoadipate]. Following exposure to elevated fluoride concentrations, the levels of six specific metabolites decreased (*P* < 0.01), whereas the levels of two metabolites increased (Fig. 9I). GY007 supplementation significantly mitigated this trend (*P* < 0.05; Fig. 9I). KEGG pathway enrichment analysis indicated that enriched pathways included lysine degradation, riboflavin metabolism, biosynthesis of cofactors, and vitamin digestion and absorption in both comparisons (Ctrl vs F and F vs F + Bi; *P* < 0.05; Fig. 9K, L). These results indicated that fluoride exposure altered the renal metabolic profile, and GY007 treatment mitigated these changes.

## Discussion

This study investigated the association between fluoride exposure from drinking water and renal injury, and provided evidence that GY007 supplementation may mitigate fluoride-induced kidney damage. Fortified foods (e.g., fluoridated salt) and drinking water are commonly considered jointly when assessing total fluoride intake (WHO 2022). To prevent excessive fluoride intake, the use of fluoride-fortified products (e.g., fluoridated salt) is often adjusted or discontinued according to local fluoride concentrations in drinking water, thereby reducing systemic fluoride burden (Tamayo-Cabeza et al. 2024). Soaking or cooking foods in fluoridated water may increase dietary fluoride intake, underscoring the need to consider cooking practices when estimating total fluoride intake from food and drinking water (Sawangjang and Takizawa 2023). Future research should assess multi-route fluoride exposure (including drinking water and diet) and test whether combined policy measures and probiotic interventions can reduce fluoride-related health risks.

Experimental studies have shown that high fluoride exposure induces renal histopathological changes and impairs kidney function in mice and rats (Wang et al. 2020a). Li et al. (2021) reported that exposure to 100 mg/L sodium fluoride induced renal biochemical alterations and dysfunction in mice, as indicated by increased BUN and Scr levels. In acute and chronic kidney disease in humans, as well as in rodent models, impaired glomerular filtration and tubular reabsorption are associated with elevated LCN2 and β2-MG levels (Marques et al. 2023; Yuan and Jin 2023). Notably, we did not observe significant differences in renal function markers between the F-Low and F-High groups. Zheng et al. (2024) reported that five months of fluoride exposure led to a dose-dependent increase in Scr, whereas BUN did not differ significantly across doses. Given that the drinking-water fluoride concentrations used here (25 and 50 mg/L) exceed the reported range of blood-water proportionality (≤ 10 mg/L; WHO 2004), a non-linear or plateauing response in internal dose or downstream injury markers with prolonged exposure is plausible. Further linear regression analysis provided additional evidence supporting this.

The rationale for ileal metagenomic analysis in Experiment 1 was to identify *Bifidobacterium* species that may confer protection against fluoride-induced kidney injury by comparing ileal species profiles between control and fluoride-exposed mice. Miao et al. reported that intestinal *Lactobacillus johnsonii* abundance decreased across different CKD stages and was positively associated with eGFR. In addition, *Lactobacillus johnsonii* supplementation alleviated renal lesions in a rat model of CKD (Miao et al. 2024). In this study, the relative abundance of *Bifidobacterium* did not differ appreciably among treatment groups when assessed at the phylum (Actinobacteria), class (Actinomycetia), and order (Actinomycetales) levels. These findings underscore that the complexity of metagenomic datasets may obscure functionally relevant microbial dynamics when analyses are limited to higher-level taxonomic categories (Gilbert et al. 2016). The results of Experiment 2, in which *Bifidobacterium animalis subsp. animalis* GY007 was used, further confirmed this finding.

Body weight changes reflect the overall health status of animals. We observed reduced body weight following fluoride exposure, consistent with prior reports of systemic toxicity (Sun et al. 2025). The differences in the kidney-to-body weight ratio among the three groups in this study may be primarily driven by variations in body weight. This pattern does not rule out renal injury, as toxicological assessment relies on integrated evidence (Avila et al. 2020; Ennulat et al. 2018). Further histological assessment and renal function indices revealed treatment-dependent alterations in kidney structure and function. Based on toxicological evidence, molecular and histopathological changes may precede alterations in gross morphology (Polascik et al. 2002). This may be attributable to differences in the mechanisms of renal injury induced by different methods, and/or to the stage of renal injury. Martinelli et al. (2025) reported that absolute kidney weight did not change significantly in a high-fat diet-induced model, despite evidence of renal injury, including glomerulosclerosis, fibrosis, and inflammation. In the study by Gelen et al. (2022), neither acrylamide-induced nephrotoxicity nor its attenuation by naringin was captured by absolute kidney weight, despite significant differences in body weight and the kidney-to-body weight ratio among treatment groups.

Oxidative stress, mitochondrial dysfunction, and inflammation may contribute to fluoride-induced nephrotoxicity (Sharma et al. 2023; Zheng et al. 2024). Administration of sodium fluoride induced a dose- and time-dependent decline in renal SOD and GSH levels in juvenile carp, whereas MDA levels increased in a dose- and time-dependent manner (Chen et al. 2015). Similarly, 600 ppm sodium fluoride significantly reduced renal GSH levels and CAT and SOD activities in male Wistar rats (Nabavi et al. 2012). Xin et al. reported that *Lactobacillus johnsonii* partially restored fluoride (100 ppm)-disrupted renal MDA, SOD, CAT, and GSH levels, although they remained significantly different from controls (Xin et al. 2021a). Oxidative stress responses to fluoride exposure are not fully consistent across studies, likely due to differences in experimental models and species. Additional evidence, particularly from human studies, is needed.

Excessive reactive oxygen species production is accompanied by increased pro-inflammatory mediators (Farooq et al. 2019) and has significant effects on renal pathophysiological processes (Foresto-Neto et al. 2024; Joyce et al. 2017). IL-6 and TNF-α are commonly used inflammatory markers to assess inflammatory status (Yu et al. 2022). It has been reported that adult male Wistar rats exposed to 15 mg/L NaF for 14 days show significantly elevated renal TNF-α levels (Owumi et al. 2019). Furthermore, ICR mice exposed to sodium fluoride (12 ppm for 6 weeks) showed increased renal expression of TNF-α and IL-6, accompanied by decreased IL-10 (Luo et al. 2017). Three *Bifidobacterium* strains reduced IL-6 and TNF-α levels in a mouse model of AKI (Meng et al. 2024). GY007 showed the regulatory potential for abnormal changes in inflammatory cytokines in the kidneys following fluoride exposure.

Ultrastructural analysis revealed mitochondrial structural damage as a hallmark of fluoride-induced renal injury in mice. The kidney has a high energy demand for glomerular filtration and tubular reabsorption, making mitochondria crucial for renal function (Ma et al. 2022). The mitochondrial oxidative phosphorylation system, which forms the MMP, provides the driving force for ATP synthesis (Xin et al. 2023). An imbalance in mitochondrial fission and fusion disrupts mitochondrial dynamics and may contribute to kidney injury (Zhang et al. 2021; Zhu et al. 2023). Drp1 is a key regulator of mitochondrial fission (Horn et al. 2020) and is upregulated in mice with chronic renal failure (Sun et al. 2020b). Downregulation of Drp1 preserves mitochondrial morphology, reduces oxidative stress, and attenuates progressive renal injury (Perry et al. 2018). In addition, Drp1-mediated mitochondrial oxidative stress has been implicated in cadmium-induced renal necroinflammation in rats (Lian et al. 2025). Fis1 is a Drp1 receptor on the mitochondrial outer membrane, anchored by a C-terminal transmembrane domain; its upregulation promotes mitochondrial fission in the kidney (Qin et al. 2015). Fluoride exposure has been reported to increase renal Fis1 expression, thereby enhancing mitochondrial fission, triggering renal tubular epithelial cell apoptosis, and contributing to nephrotoxicity (Zuo et al. 2024). Song et al. reported that disrupting the Drp1-Fis1 interaction alleviates pathological mitochondrial fragmentation and acute kidney injury (Song et al. 2024). In an adenine-induced CKD mouse model, the Drp1/Fis1 axis was shown to induce mitochondrial dysfunction in the cerebral cortex, as evidenced by increased MDA and ROS levels, reduced SOD activity and ATP content, and enhanced mitochondrial fission (Sun et al. 2022). Impaired mitochondrial biogenesis is a major contributor to mitochondrial dysfunction and plays an important role in kidney disease progression (Zuo et al. 2024). Mitochondrial biogenesis can be assessed by mtDNA content (Xin et al. 2023). In our study, fluoride significantly reduced renal mtDNA content, suggesting compromised mitochondrial biogenesis. Notably, mtDNA copy number is closely associated with oxidative stress and is widely used as an indicator of mitochondrial function (Kumar et al. 2025). The oxidative phosphorylation (OXPHOS) comprises five respiratory-chain complexes containing nuclear- and mtDNA-encoded subunits. Fluoride dysregulated eight OXPHOS subunit transcripts, whereas GY007 restored only mtND3, mtcyb, mtCO2, and Sdhb. Recent studies suggest that probiotics can ameliorate mitochondrial dysfunction in non-renal settings, including Alzheimer’s disease and doxorubicin (DOX)-induced cardiotoxicity (Lin et al. 2025; Wang et al. 2025); however, evidence in the kidney remains limited. Given the frequent co-occurrence of oxidative stress, inflammation, and mitochondrial dysfunction, future studies should directly evaluate mitochondrial complex activities and ATP production to better define renal mitochondrial effects.

Renal metabolism remains incompletely characterized despite its central role in systemic metabolic regulation. Here, eight differential metabolites (VIP > 2) were common to both comparisons. Ammelide (MN1072), a melamine derivative reported in feed, water, and dust (Zhu et al. 2019; Zhu and Kannan 2018; Liu et al. 2025), was detected in kidney, suggesting environmental/dietary exposure; however, evidence for ammelide toxicity is limited (Day et al. 2024) and it has not been linked to acute nephrotoxicity (Dobson et al. 2008). Melamine-related compounds were identified in pediatric renal calculi, whereas ammelide was not detected in these specimens (Campbell et al. 2024). Given the role of the gut microbiota in melamine biotransformation, altered renal ammelide levels may reflect changes in intestinal absorption, metabolism, or clearance within a fluoride-disrupted gut-kidney axis, which may be modulated by probiotics. Aminoadipate (MP4448), a lysine-metabolism intermediate and redox-imbalance marker (Zeitoun-Ghandour et al. 2011), and the mitochondria-confined saccharopine pathway (Leandro and Houten 2020) further implicate mitochondrial involvement: saccharopine accumulation disrupts mitochondrial morphology/function (Leandro and Houten 2019) and increases markedly after renal ischemia–reperfusion injury (Beier et al. 2020). Together with our mitochondrial findings, these data suggest that lysine-metabolism disturbances may contribute to fluoride-associated mitochondrial injury. OS-PC (MP34017) has been reported to promote renal inflammation via ROS (Jiao et al. 2023), consistent with the anti-inflammatory/antioxidant effects of GY007 observed here. Fluoride also decreased N-acetyl-DL-phenylalanine and N-acetyl-L-tyrosine, which were restored by probiotics, suggesting perturbed aromatic amino acid acetylation; reduced N-acetyl-L-tyrosine may indicate blunted stress adaptation (Matsumura et al. 2020), whereas the renal decline of N-acetyl-L-phenylalanine-proposed as a microbiota-associated nephrotoxic metabolite in hyperuricemic kidney disease (Peng et al. 2025)-should be interpreted with plasma/urine levels. Finally, GY007 also affected MN11776, MP21033, and MP19911, warranting further characterization.

This study has several limitations. (1) Although the exposure dose was selected to be environmentally relevant, the model was not designed to quantify the combined contribution or real-world variability of dietary fluoride intake (e.g., from fortified foods) and background environmental exposure. Therefore, extrapolation to aggregate fluoride exposure, changes in body burden, and related public health implications should be made with caution. Future studies should incorporate multi-source exposure assessment. (2) The causal roles of the gut microbiota and kidney metabolites in fluoride-induced renal injury, as well as in probiotic-mediated protection, remain incompletely defined. Future work should employ causality-oriented approaches, such as microbiota or metabolite depletion/modulation, longitudinal microbiome-metabolome profiling following GY007 intervention, and targeted manipulation of specific microbial taxa, metabolites, or defined consortia, to clarify microbiota/metabolite-dependent mechanisms in fluoride-associated nephrotoxicity. (3) To date, there is no universally accepted mouse model for high-fluoride exposure. The doses and duration used here were informed by our prior studies (Huang et al. 2024; Sun et al. 2025; Xin et al. 2025, 2023) and published reports (Guo et al. 2020; Zheng et al. 2024; Singh 2023). Although the exposure models produced marked renal toxicity, future studies should further evaluate the biological relevance of the selected sodium fluoride dose and exposure duration. (4) While GY007 showed renoprotective effects, the intervention window in Experiment 2 was limited; thus, its durability in the chronic drinking-water model should be validated in longer-term studies.

## Conclusion

Prolonged intake of drinking water with environmentally relevant fluoride levels induces renal injury in mice. Fluoride exposure reduced the abundance of *Bifidobacterium* in the ileum, which was significantly and negatively correlated with kidney injury markers. Administration of *Bifidobacterium animalis* GY007 attenuated renal damage induced by high-fluoride exposure (24 mg/kg/day for 8 weeks). The renoprotective effects of GY007 may be attributed to enhanced antioxidant capacity, suppression of inflammation, and partial restoration of mitochondrial homeostasis. Metabolomics further identified eight metabolites associated with GY007-mediated renal recovery. Nevertheless, the precise mechanisms underlying these nephroprotective effects warrant further investigation. Collectively, our findings support the development of probiotic interventions to protect populations exposed to high fluoride levels in drinking water.

## Supplementary Information

Below is the link to the electronic supplementary material.

Supplementary Material 1.

Supplementary Material 2.

## Data Availability

The metagenome sequencing data of ileum has been submitted to the Genome Sequence Archive (PRJCA049427).
