# Supplementary material for: Targeting Bifidobacterium animalis alleviates high-fluoride exposure-induced kidney injury in mice
Source: AMB Express. 2026 Apr 8;16:59. doi: 10.1186/s13568-026-02031-7 (PMC13194810; doi:10.1186/s13568-026-02031-7)
Supplement: Supplementary file 2 — Supplementary Material 2. [file 13568_2026_2031_MOESM2_ESM.docx]

Supplementary Material

**Figure**

**
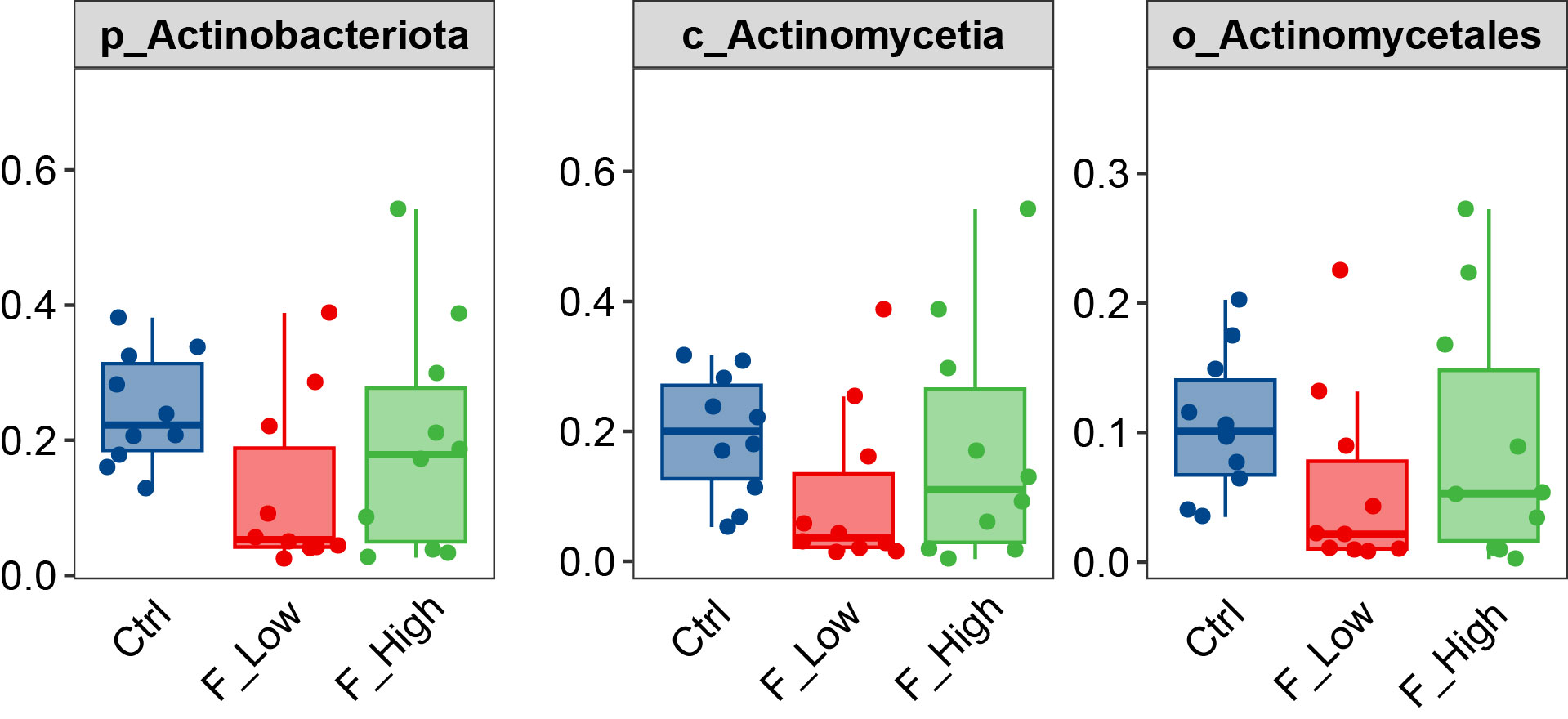
**

**Figure S1 The relative abundances of p_Actinobacteriota, c_Actinomycetia and o_Actinomycetales in controls and two groups of mice exposed to different fluoride concentrations.**

**Table**

**Table S1 Primer sequences for RT-qPCR experiment**

| **Gene** | **Forward (5′→3′)** | **Reverse (5′→3′)** |
| --- | --- | --- |
| β-actin | GCTCTTTTCCAGCCTTCCTT | GATGTCAACGTCACACTT |
| 18s rRNA | GTAACCCGTTGAACCCCATT | CCATCCAATCGGTAGTAGCG |
| IL-6 | TGCAAGAGACTTCCATCCAGT | GTGAAGTAGGGAAGGCCG |
| IL-10 | GGTTGCCAAGCCTTATCGGA | ACCTGCTCCACTGCCTTGCT |
| TNF-α | ACGGCATGGATCTCAAAGAC | AGATAGCAAATCGGCTGACG |
| mtND1 | TCCTATCCACGCTTCCGCTA | GGTGGTATCCCCGCTGTAA |
| mtND3 | TGCACGCCTACCATTCTCAA | GGTAGTGGAAGTAGAAGGGCA |
| mtND4L | AGCCTTCTCACTGTCACTCC | GGCTAGTCCTACAGCTGCTT |
| NDUFB8 | GGTSTGGCGACTACCCGATG | GGCTCAGGCTCTTTGGTAGG |
| Sdhb | CTACGTTCATGGGGACACCC | CAGATGCCGACATCGTGGTA |
| mtcyb | TTTATCATCGCGGCCCTAGC | ATGGGGTGGGGTGTTTAGTG |
| UQCRC2 | GGTTAAAACTTCAGCAGCCCC | TGCTGTGGTGACATTGAGCA |
| mtCo2 | ATAACCGAGTCGTTCTGCCA | CCTGGTCGGTTTGATGCTACT |
| mtAtp6 | ACACACCAAAAGGACGGACA | AGTGGGCGAGTGAGCTTTTT |
| Kim-1 | GGGTCTCCTTCACAGCACATT | CAAGGCCAGCCCTCTAATGG |

**Materials and methods**

**Rationale for fluoride exposure levels**

The FDA suggests (FDA 2005) that a human equivalent dose (HED) in drug exposure studies is:

[HED = Animal dose multilied by (*Animal Km*/*Human Km*)] .

Where *Km* values are the ratio of body weight to surface area (*mouse Km* = 3, *Human Km* = 37). This equation has also been used in a study about arsenic exposure (Coryell et al. 2018). In Experiment 1, using this equation, 25 and 50 ppm fluoride exposure in mice correspond to HEDs of 2.03 ppm and 4.05 ppm. In Experiment 2, using this equation, 24 mg/kg fluoride exposure in mice correspond to HEDs of 1.95 mg/kg. For an adult weighing 55 kg and with a daily water intake of 4.5 L, it is 23.8 ppm.

Coryell M, McAlpine M, Pinkham NV, McDermott TR, Walk ST (2018) The gut microbiome is required for full protection against acute arsenic toxicity in mouse models. Nat Commun 9(1):5424 doi:10.1038/s41467-018-07803-9

U.S. Food and Drug Administration (2005) Guidance for Industry: Estimating the Maximum Safe Starting Dose in Initial Clinical Trials for Therapeutics in Adult Healthy Volunteers. Center for Drug Evaluation and Research (CDER), July 2005. https://www.fda.gov/media/72309/download. Accessed 21 Jan 2026.

**Metagenomic analysis and bioinformatics analysis**

Using OMEGA Mag-Bind DNA Kit (M5635-02; Omega Bio-Tek, Norcross, GA, USA) to extract the genomic DNA of all microorganisms, completely according to the steps in the instructions. The extracted DNA was stored at -20°C for later analysis. The concentration of DNA was measured using a Qubit™ 4 Fluorometer (Q32856 and Q33231), and the completeness of DNA was checked by agarose gel electrophoresis. In order to construct a metagenomic shotgun sequencing library with an insertion fragment of approximately 400 bp, the extracted microbial DNA was first treated with the Illumina TruSeq Nano DNA LT Library Preparation Kit. Subsequently, each library was sequenced at Personal Biotechnology Co., Ltd. using the Illumina NovaSeq platform with a paired-end 150 bp method. To obtain high-quality data for further analysis, the original sequencing data were processed using Cutadapt (v1.2.1; Martin, 2011), the sliding-window algorithm in fastp (v0.23.2; Chen et al., 2018), and Minimap2 (v2.24-r1122; Li, 2018). The metagenomic sequencing data of all samples were classified and analyzed using GTDB as a reference database. The reads classified as metazoans or viridiplantae in the following analysis were not used in this study. The reads of each sample were assembled using Megahit version 1.1.2 (Li et al., 2015) with default meta-large settings. Sequences longer than 300 base pairs were combined and grouped using a tool called MMseqs2 (Steinegger and Söding, 2017). This method uses the 'easy-linclust' algorithm, which requires sequence similarity to reach 95% and at least 90% coverage of each contig. The unique contigs were compared with the NCBI-nt database using MMseqs2 in ‘taxonomy’ mode to determine their lowest common ancestor classification. All contigs belonging to Viridiplantae or Metazoa were removed. Genes in the contigs were predicted using Prodigal software (version 2.6.3; Hyatt et al., 2010). The coding sequences (CDS) in all samples were divided using the 'easy-cluster' mode of MMseqs2. The standard is that the protein sequence similarity must reach 0.95, and at least 90% of the short contig must be covered. High-quality reads from each sample were aligned to the predicted gene sequences using Minimap2 with the parameters ‘-ax sr -sam-hit-only’. The number of reads mapped to each gene sequence was then quantified using featureCounts, with the resulting value referred to as ‘Read Count (RC)’. To observe the differences in microorganisms among the various groups, the LEfSe (linear discriminant analysis effect size) method with default settings was used (Segata et al., 2011). Alpha diversity indices, including Chao1, Shannon, and observed species, were calculated and visualized using box plots. Beta diversity analysis, based on the Bray-Curtis distance metric, was performed to investigate structural variations in microbial community composition across different groups and subsequently visualized through principal coordinate analysis (PCoA). Spearman rank correlations were calculated and visualized as a heatmap using the R package psych.

Martin MJEJ (2011) Cutadapt removes adapter sequences from high-throughput sequencing reads. J Comput Biol 17(1) 1138-1143. doi: 10.1089/cmb.2017.0096

Chen S, Zhou Y, Chen Y, Gu J (2018) fastp: an ultra-fast all-in-one FASTQ preprocessor. Bioinformatics 34(17):i884-i890 doi:10.1093/bioinformatics/bty560

Li H (2018) Minimap2: pairwise alignment for nucleotide sequences. Bioinformatics 34(18):3094-3100 doi:10.1093/bioinformatics/bty191

Li D, Liu CM, Luo R, Sadakane K, Lam TW (2015) MEGAHIT: an ultra-fast single-node solution for large and complex metagenomics assembly via succinct de Bruijn graph. Bioinformatics 31(10):1674-6 doi:10.1093/bioinformatics/btv033

Steinegger M, Söding J (2017) MMseqs2 enables sensitive protein sequence searching for the analysis of massive data sets. Nat Biotechnol 35(11):1026-1028 doi:10.1038/nbt.3988

Hyatt D, Chen GL, Locascio PF, Land ML, Larimer FW, Hauser LJ (2010) Prodigal: prokaryotic gene recognition and translation initiation site identification. BMC bioinformatics 11:119 doi:10.1186/1471-2105-11-119

Segata N, Izard J, Waldron L, Gevers D, Miropolsky L, Garrett WS, Huttenhower C (2011) Metagenomic biomarker discovery and explanation. Genome Biol 12(6):R60 doi:10.1186/gb-2011-12-6-r60

**Biochemical detection**

The levels of dynamin-related protein 1 (Drp1), fission 1 (Fis1) in the kidney, β2-microglobulin (β2-MG) and lipocalin 2 (LCN2) in the serum and urine were assayed using ELISA commercial kits (Jiangsu Enzyme Technology, China). Serum blood urea nitrogen (BUN; BC1535) and creatinine (Scr; BC4915) levels were determined using commercial assay kits (Solarbio Science and Technology, Beijing, China). Antioxidant parameters in renal tissue were quantified with kits obtained from Nanjing Jiancheng Bioengineering Institute (Nanjing, Jiangsu, China), including catalase (CAT; A007-1-1), total antioxidant capacity (T-AOC; A015-3-1), malondialdehyde (MDA; A003-1-2), hydrogen peroxide (H₂O₂; A064-1-1), reduced glutathione (GSH; A006-2-1), and superoxide dismutase (SOD; A001-3-2).

Figure 1. (B) The β2-MG of urine:Wilcoxon rank-sum test. The remaining: One-way ANOVA.

Figure 3. (B-C) Wilcoxon rank-sum test.

Figure 5. (A-F) The β2-MG of urine: Wilcoxon rank-sum test. The remaining: One-way ANOVA.

Figure 6. (A-F) The GSH, SOD, and MDA: Wilcoxon rank-sum test. The remaining: One-way ANOVA.

Figure 7. (A-C) One-way ANOVA.

Figure 8. (A) Wilcoxon rank-sum test. (B-I) mtND4L and NDUFB8: Wilcoxon rank-sum test. Others: One-way ANOVA.
